# Multi-modal circulating cell-free DNA profiling to predict response to docetaxel in metastatic castration-resistant prostate cancer

**DOI:** 10.1038/s41698-026-01454-6

**Published:** 2026-04-28

**Authors:** David D. Chen, Anat Zimmer, David D. Yang, Edoardo Francini, Robert Patton, Jett Crowdis, Pooja Chandra, Irbaz Bin Riaz, Brian Hanratty, Micah Rickles-Young, Junko Tsuji, Carrie Cibulskis, Mark Fleharty, Bridget Whelpley, Brendan Reardon, Jihye Park, Peter S. Nelson, Franklin W. Huang, Eliezer M. Van Allen, Gavin Ha, Atish D. Choudhury

**Affiliations:** 1 Fred Hutchinson Cancer Center, Seattle, WA USA; 2 University of Washington, Seattle, WA USA; 3 Brigham and Women’s Hospital, Boston, MA USA; 4 Dana-Farber Cancer Institute, Boston, MA USA; 5 Department of Experimental and Clinical Medicine, University of Florence, Florence, Italy; 6 Yale School of Medicine, New Haven, CT USA; 7 Mayo Clinic, Phoenix, AZ USA; 8 Broad Institute of MIT and Harvard, Cambridge, MA USA; 9 Boston University, Boston, MA USA; 10 University of California, San Francisco, San Francisco, CA USA

**Keywords:** Biomarkers, Cancer, Computational biology and bioinformatics, Oncology

## Abstract

There are currently no clinically validated markers for taxane sensitivity in metastatic castration-resistant prostate cancer (mCRPC), so we aimed to predict docetaxel response from circulating cell-free DNA. We identified 180 patients with pre-treatment plasma specimens collected within 12 months of starting docetaxel for mCRPC at our institution. 138 underwent ultra-low pass whole genome sequencing (ULP-WGS), and tumor fractions (TFx) and copy number alterations (CNAs) were derived using ichorCNA. 79 samples with TFx > 0.04 underwent targeted panel sequencing (TPS). *TP53* mutation was significantly associated with docetaxel non-response (*p* = 0.018); deletions involving bands located in arms 11p, 11q, 10q and 3p were enriched in responders, and amplifications in regions of 1p and 6q were enriched in non-responders. Transcription factor (TF) binding activity was inferred using Griffin, which identified TFs (ZSCAN4, CTCF, PHOX2B) with trends towards increased activity in non-responders (*n* = 22) and others (including PBX1, MYBL2, OSR2, PDX1 and ZIC2) in responders (*n* = 24). A combined ensemble binary classifier generated through XGBoost integrating these feature sets to predict docetaxel response outperformed models derived from any single feature set, achieving a training area-under-the-ROC curve of 0.87. Pre-cabazitaxel specimens, representing a docetaxel-resistant population, were used for external validation, with a concordance of 79.6% for predicting non-response.

## Introduction

As treatment options for advanced prostate cancer continue to expand, optimizing treatment selection is of critical importance to maximize clinical benefit and maintain quality of life^1,2^. Treatment with an ineffective agent not only subjects the patient to adverse effects of futile therapy but also allows for progression of cancer symptoms and may limit candidacy and benefit from subsequent therapies. Indeed, real-world data demonstrates significant attrition in the number of patients with metastatic castration-resistant prostate cancer (mCRPC) who receive subsequent lines of treatment after progression^3,4^. Molecular and genetic features have been utilized for treatment selection in mCRPC^5,6^. including predicting response to PARP inhibitors^7,8^, or the immune checkpoint inhibitor pembrolizumab^9,10^. Although the chemotherapeutic agent docetaxel is a standard of care treatment for both metastatic hormone-sensitive prostate cancer and mCRPC, there are no clinically validated predictors of taxane response. Certain molecular features, such as loss of tumor suppressor genes^11,12^ or high-risk gene expression profiles^13,14^ have been previously suggested as potential predictors of taxane efficacy. However, they seem to be primarily prognostic of poor clinical outcomes with hormonal manipulations alone, and may not necessarily predict sensitivity to taxanes compared to other potentially active agents in this setting^15–19^.

Novel analytic tools for circulating cell-free DNA (cfDNA) sequencing data are emerging as promising methods to identify biomarkers for treatment selection without the need for an invasive biopsy. These tools may allow for phenotypically characterizing a patient’s tumor burden beyond activating or inactivating mutations in specific genes^20^. For example, homologous recombination repair or mismatch repair (MMR) deficiency can be detected using genomic scar-based classifiers from cfDNA sequencing, even when a causal DNA repair gene mutation is not detected^21–23^.

Previously, we described a computational tool called ichorCNA that allows the assessment of tumor fraction (TFx) and copy number alterations (CNAs) from relatively low-cost ultra-low pass whole genome sequencing (ULP-WGS), overcoming the need for deep sequencing, which can be prohibitively expensive for routine clinical care^24^. Using this tool, we demonstrated that changes in TFx are associated with treatment response and progression in prostate cancer^25^ and that CNAs (including non-focal amplifications and deletions that would not be fully characterized through targeted sequencing) are prognostic in triple-negative breast cancer^26^. In addition, the fragmentation pattern of the cfDNA—so-called “fragmentomics”^27,28^—can be used for cancer detection and identifying tissue-of-origin^29,30^, as well as for inferring gene expression^31,32^. In this regard, we developed Griffin, a tool to infer transcription factor binding activity based on fragmentation patterns in cfDNA^33^, which has demonstrated utility in distinguishing neuroendocrine prostate cancer from prostate adenocarcinoma^34^.

We previously demonstrated in an institutional cohort of mCRPC patients treated with docetaxel or cabazitaxel that ULP-WGS from banked plasma specimens is feasible and can identify genes with CNAs that are enriched in patients with cancers that are responsive or display intrinsic resistance to treatment^35^. We hypothesized that a combined multi-omic approach could identify additional genomic features from genetic profiling of cfDNA that may allow for predicting response or resistance to docetaxel.

In this study, we performed both targeted panel sequencing and ULP-WGS on cfDNA from plasma samples collected prior to starting docetaxel for mCRPC and assessed how the association of mutations detected by targeted sequencing, as well as CNAs and imputed transcription factor binding from ULP-WGS, may correlate with response or intrinsic resistance to docetaxel. We then tested whether a classifier combining these features could accurately predict docetaxel response or resistance to assess the potential clinical utility of multi-modal cfDNA profiling.

## Results

### Patient characteristics

**Fig. 1:**
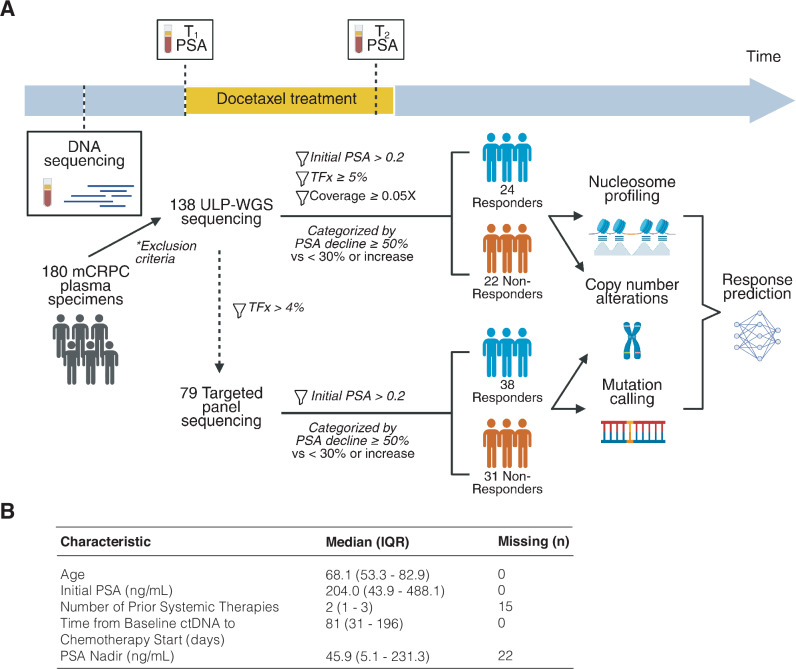
Study design and patient stratification. **A** Schematic of the patient selection and sequencing workflow. Blood samples from patients with metastatic castration-resistant prostate cancer (mCRPC) underwent ultra-low pass whole genome sequencing (ULP-WGS) and/or targeted panel sequencing. Samples were selected for ULP-WGS based on the inclusion criteria detailed in the Results; those with adequate tumor fraction (TFx) and genome coverage underwent nucleosome profiling, and those with TFx > 0.04 underwent targeted panel sequencing. Patients with baseline PSA < 0.2 ng/ml (*n* = 2) and those with intermediate PSA responses (i.e., a PSA decline between 30 and 50%, *n* = 8) were excluded from responder/non-responder analysis. **B** Clinical characteristics of the pre-docetaxel mCRPC cohort, including baseline PSA levels and post-treatment PSA responses.

We identified 180 patients with metastatic prostate cancer who had blood samples collected within 12 months prior to initiation of docetaxel (Fig. 1A), of whom 138 underwent ULP-WGS. Reasons for exclusion were the following: docetaxel administered in HSPC or biochemically recurrent disease (*n* = 14), clinical efficacy data unavailable (*n* = 18), docetaxel administered in combination (*n* = 8), and plasma specimens not located (*n* = 2). Of these, 79 samples had TFx > 0.04 by ichorCNA and underwent targeted panel sequencing. Baseline characteristics of the cohort are summarized in Fig. 1B. The median initial prostate-specific antigen (PSA) level across samples was 204.0 ng/mL (interquartile range [IQR]: 43.9–488.1). Patients had received a median of 2 prior systemic therapies (IQR: 1–3), with data missing for 15 patients. The median time between circulating cfDNA collection and docetaxel initiation was 81 days (IQR: 31–196). The median PSA nadir was 45.9 ng/mL (IQR: 5.1–231.3), with data missing for 22 patients.

### *TP53* mutations are more common in patients who do not respond to docetaxel treatment

**Fig. 2:**
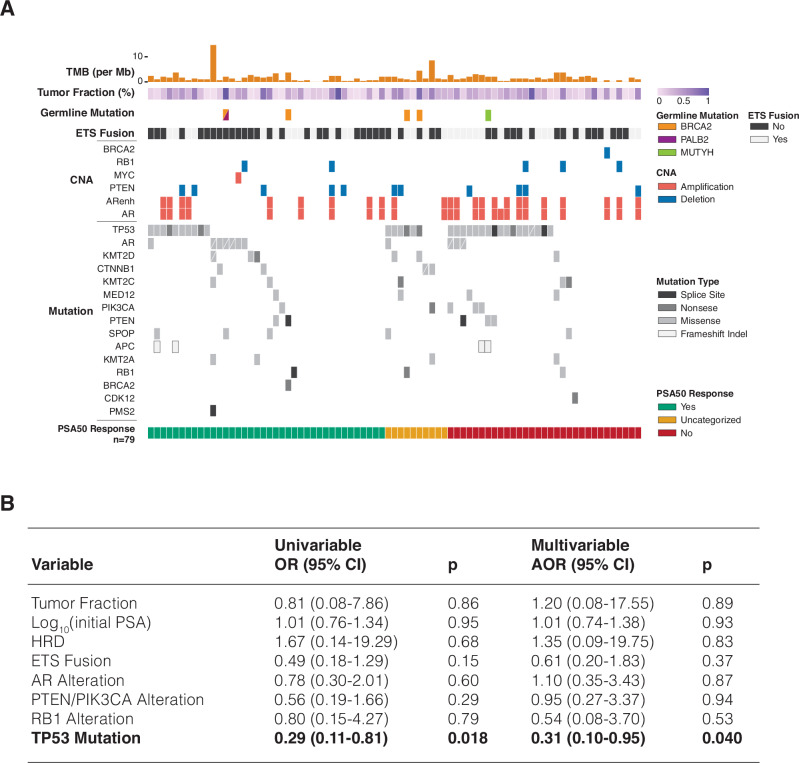
Genomic alterations in mCRPC and their association with PSA50 response. **A** Comutation plot depicting genomic alterations identified in 79 patients with metastatic castration-resistant prostate cancer (mCRPC) included in the study cohort. Circulating tumor DNA (ctDNA) analysis revealed several pathogenic or likely pathogenic germline mutations, including in *BRCA2* (*n* = 4), *PALB2*, and *MUTYH*. Somatic mutations are shown for genes reported by Armenia et al.^37^, alongside copy number gains and deletions annotated based on the described criteria, as well as ETS fusions identified using SVAbA. **B** Logistic regression analysis of PSA50 response was performed on 69 evaluable patients. Inclusion criteria required a baseline PSA ≥ 0.2 ng/mL prior to initiating docetaxel and excluded patients with an intermediate PSA response (i.e., a PSA decline ≥30% but <50%). *TP53* mutation was significantly associated with reduced odds of achieving a PSA50 response.

Somatic mutations were analyzed as previously described^36^ with a focus on genes included in the “long tail” of significantly mutated genes identified in Armenia et al.^37^ and four MMR genes, filtered for pathogenic variants cataloged in the COSMIC database. Key prostate cancer-related pathways, including androgen receptor (*AR*), *PTEN/PIK3CA*, and *RB1*, were assessed for both mutations and copy number changes. Serum cfDNA analysis identified a range of genomic alterations, including pathogenic germline mutations in *BRCA2* (four patients), *PALB2*, and *MUTYH* (Fig. 2A*)*. Additionally, copy number changes and somatic mutations (restricted to genes from the Armenia panel) as well as ETS fusions involving *TMPRSS2* and ETS family genes (e.g., *ERG*, *ETV1*, *ETV4*, *ETV5*, *ELK4*, and *FLI1*) are illustrated.

Logistic regression analyses evaluated the association between genomic alterations and the likelihood of being a docetaxel “responder,” defined as achieving a ≥ 50% decline in PSA (PSA50 response) vs. a “non-responder,” defined as not achieving a ≥ 30% decline in PSA (i.e., demonstrating intrinsic resistance to docetaxel) (Fig. 2B). Among the 69 patients with evaluable PSA data (excluding 2 patients with PSA < 0.2 before docetaxel initiation and those with intermediate responses between a 30% and 50% PSA decline), the presence of a *TP53* mutation was significantly associated with reduced odds of achieving a PSA50 response in both univariable analysis (OR 0.29; 95% CI: 0.11–0.81; *p* = 0.018) and multivariable analysis (AOR 0.31; 95% CI: 0.10–0.95; *p* = 0.040). Other variables, including TFx, initial PSA levels, homologous recombination deficiency (HRD), ETS fusions, *AR* alterations, *PTEN*/*PIK3CA* alterations, and *RB1* alterations, did not show statistically significant associations with PSA50 response.

### Docetaxel responders exhibit different copy number alterations (CNAs) compared to non-responders

**Fig. 3:**
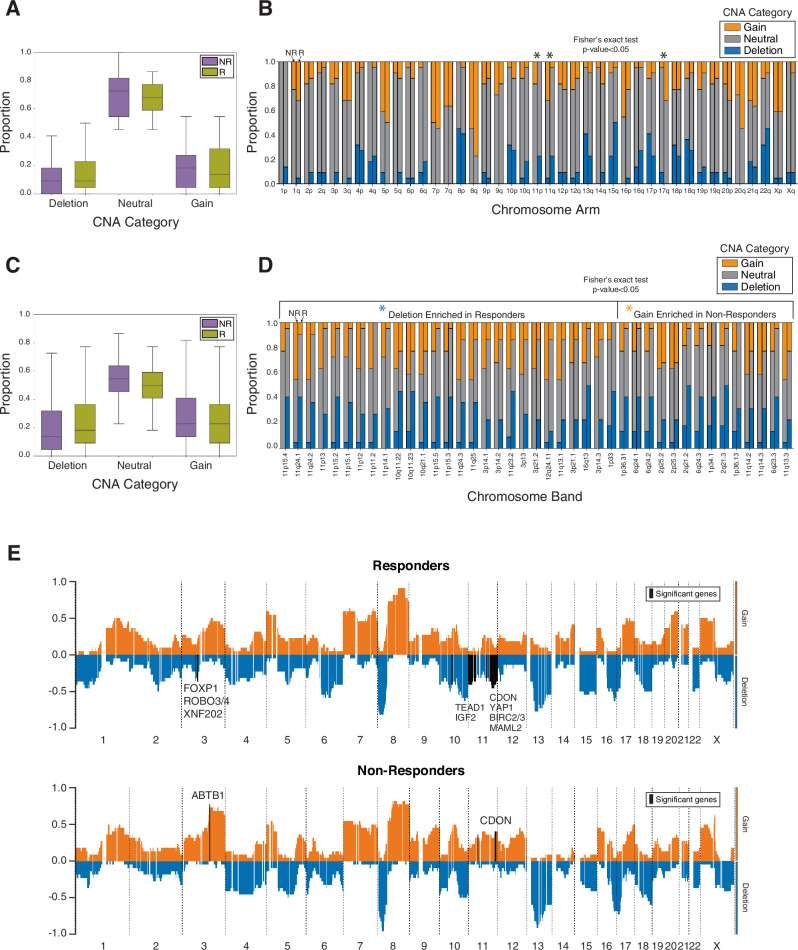
Genomic copy number alterations distinguish docetaxel responders from non-responders in mCRPC. Copy number variations were calculated using the ichorCNA software across multiple genomic levels: chromosome arm (**A**, **B**), band (**C**, **D**), gene, and bin (**E**). **A** A comparison between the three copy number ranks in responders compared to non-responders (chi-square test). **B** Chromosome Arm Copy Number: The proportions of three copy number ranks—0.5 (deletion), 1 (neutral), and 1.5 (gain)—are shown for each chromosome arm across groups (non-responders left column, responders right column). Chromosomes are ordered by the most significant differences in copy number, determined by the Fisher’s exact test (*p* values shown in Supplementary Data 1-sheet 2). **C**, **D** Band-Level Analysis: similar to **A**, **B**, but the comparison is at the band level instead of the arm level (*p* values shown in Supplementary Data 1-sheet 3). **E** Bin-Level Overview: a complete view of copy number variations across all chromosomes, with amplifications shown in orange and deletions in blue. Bins with (nominally) statistically significant differences in responders vs. non-responders are depicted in black, and selected genes within these bins are depicted below the bins.

Using ichorCNA^24^, we assessed CNAs from ULP-WGS in responders and non-responders across three genomic scales: chromosome arms, bands, and genes. Overall, we observed instances of different alteration frequencies between response groups at all scales, although none reached statistical significance after multiple hypothesis testing. At the chromosome arm and band levels, we observed no statistical significance in the overall proportion of CNAs between responders and non-responders (Fig. 3A, C). While there was no significance after multiple hypothesis correction, we observed deletions of 11p and 11q and gains of 17q to be enriched in responders compared to non-responders (Fisher’s exact test, *p* < 0.05; Fig. 3B, Supplementary Data 1-sheet 2). When evaluating CNA events affecting cytoband regions, we observed trends towards a higher proportion of copy number deletions in 33 cytobands in responders and gains in 18 cytobands in non-responders (Fisher’s exact test, *p* < 0.05, Fig. 3D, Supplementary Data 1-sheet 3). The majority of cytobands enriched for deletions were on chromosome 11 (23 bands), while amplifications were enriched predominantly on chromosomes 11 (10 bands) and 12 (4 bands).

We identified 646 deleted and 142 amplified genes that were nominally enriched in responders compared to non-responders (Chi-squared test, *p* < 0.05, Fig. 3E, Supplementary Data 1-sheets 4 and 5). The majority of genes enriched for deletions in responders were localized to chromosome 11, followed by chromosomes 3 and 10, while genes enriched for amplifications in non-responders predominantly occurred on chromosomes 3 and 11 (Fig. 3E). Among genes in the Hippo-YAP, PI3K-Akt, and Notch signaling pathways (765 genes) that have previously been implicated in docetaxel resistance^38–40^, 17 exhibited differential CNAs (1 gain, 16 deletions), including gain/loss of *CDON* and loss of *YAP1* and *TEAD1* (Supplementary Data 1-sheets 6 and 7). Overall, tumor fraction and ploidy determined by ichorCNA were not significantly different between the groups (*p* = 0.62 and *p* = 0.97, respectively, Supplementary Fig. 1A).

### Distinct inferred transcription factor binding activity and chromatin accessibility patterns between responders and non-responders

**Fig. 4:**
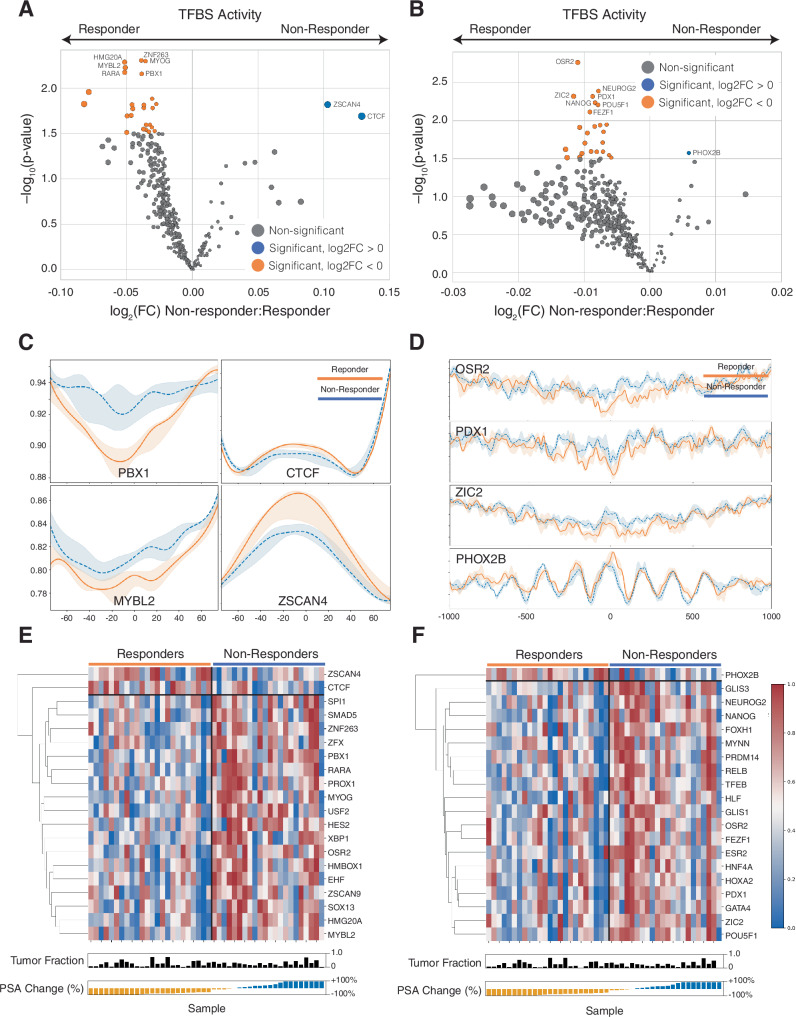
Nucleosome profiles reveal transcription factor activity differences associated with docetaxel response. A list of 377 common transcription factors (TFs) was compiled, and nucleosome profiles for 10,000 GTRD binding sites were aggregated for each TF. Using Griffin, an average profile for each TF was generated. The average differences in central coverage (sequencing coverage at the midpoint of the profile, see “Methods”) were then calculated. **A** Of the 377 TFs, 56 exhibited significant differences in central coverage, of which 54 were more active in responders compared to non-responders, while CTCF and ZSCAN4 exhibited the opposite trend, showing greater activity in non-responders. HMG20A showed the largest difference between groups. **B** 37 TFs exhibited significant differences in mean coverage, with all except PHOX2B being more active in responders compared to non-responders. **C** A zoomed-in view of the central region for four exemplary TFs. **D** Full profiles of four selected TFs: OSR2—exhibited the largest difference in mean coverage, with greater activity in responders. **E**, **F** Heatmaps showing central and mean coverage values, respectively, for the 56 significant TFs (rows) across 24 responders (orange line) and 22 non-responders (blue line). Quantile normalization was applied.

Analysis of cfDNA fragmentation patterns can reveal transcription factor (TF) binding activity of the tumor through inference of nucleosome occupancy at transcription factor binding sites (TFBSs)^33,41–43^. We applied the Griffin tool to analyze the nucleosome accessibility surrounding 10,000 TFBSs for each of 377 TFs (Methods). For each TF, we generated an aggregated, composite nucleosome profile by combining the 10,000 TFBSs. The central (±30 bp) and mean (±1000 bp) coverages were extracted from the composite profile for each TF. Lower coverages indicate increased accessibility and thus, increased TF binding activity. We identified TFs that were enriched for activity in responders and non-responders for central coverage (Fig. 4A) and mean coverage (Fig. 4B), although none were significant after false discovery rate (FDR) multiple testing correction. Nevertheless, we observed notable trends of increased inferred activity in non-responders, such as for ZSCAN4, CTCF, and PHOX2B. By contrast, activity was higher in responders for 25 TFs by central coverage, including HMG20A, PBX1, and MYBL2 (Fig. 4C, Supplementary Data 1-sheet 8), and 24 TFs by mean coverage, including OSR2, PDX1, and ZIC2 (Fig. 4D, Supplementary Data 1-sheet 9). Overall, we observed a higher number of TFs having increased inferred activity in responders compared to non-responders for both central (Fig. 4E) and mean (Fig. 4F) coverage metrics.

To evaluate if chromatin accessibility inferred from cfDNA can distinguish the docetaxel response status, we examined model system data for orthogonal assessment of this hypothesis. Specifically, we first identified regions of differentially accessible chromatin using ATAC-seq data from patient-derived xenografts (PDX) tumor tissue (Methods). We identified a total of 3872 regions with differential activity between responders and non-responders (*p* < 0.05), which comprised 2247 regions with increased activity in responders (“responder-specific sites”) and 1625 regions with increased activity in non-responders (“non-responder-specific sites”). Using TritonNP at these differential regions, we analyzed the nucleosome coverage to infer accessibility (decreased coverage) and inaccessibility (increased coverage) in the 46 cfDNA samples. We observed a trend of increased accessibility in responders for the responder-specific sites (Supplementary Fig. 2A). There was no difference between the responder and non-responder groups in the non-responder-specific sites (Supplementary Fig. 2B).

### Multi-omic integration enhances the prediction of docetaxel treatment response compared to single-approach analyses

**Fig. 5:**
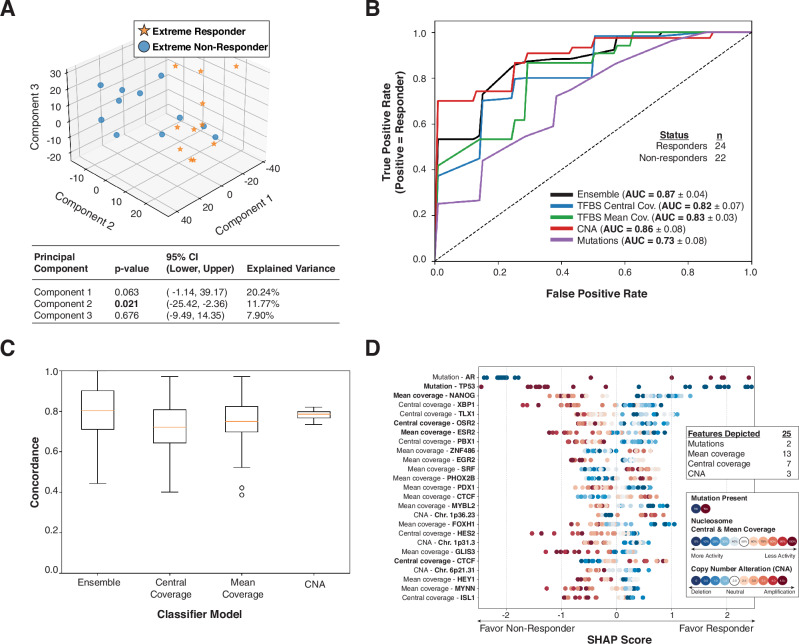
Classification of docetaxel response. **A** Top three principal components plotted for the 11 strongest responders and 11 strongest non-responders based on post-treatment PSA change using combined features (total *N* = 1461) of central coverage, mean coverage, copy number alterations, and selected mutations. **B** XGBoost Classifier results for predicting response to docetaxel treatment for 46 mCRPC patients, showing area-under-the-ROC curves when trained on mean coverage, central coverage, copy number alterations, selected mutation, and a combined ensemble model. The ensemble model was validated in (**C**) using a hold-out approach across 100 iterations to predict cabazitaxel response (*N* = 24, all non-responders) with plotted concordance index for predicting treatment failure or non-response. **D** Plot of SHapley Additive exPlanations (SHAP) scores, selecting the top 25 most impactful features for affecting the ensemble model performance when weighing the probability of docetaxel responder vs non-responder status.

We observed that cfDNA features based on nucleosome coverage (central and mean) for 377 TFs, CNAs, and mutations could distinguish between “extreme” responders (post-treatment PSA decrease >80%) and non-responders (post-treatment PSA increase >30%) in the cohort (Fig. 5A). Therefore, we developed a machine learning model to predict the response to docetaxel from pre-treatment plasma cfDNA using these as independent feature sets. We conducted cross-validation using XGBoost on ULP-WGS cfDNA data from 46 pre-treatment mCRPC patients (Methods). The model produces a binary prediction to indicate whether a sample is likely a responder or at risk of being a non-responder to docetaxel treatment. The training performance by cross-validation was computed using the mean area under the receiver operating characteristic curve (AUC) for each feature set individually: 0.82 (central coverage for TFs), 0.83 (mean coverage for TFs), 0.86 (CNAs), and 0.73 (mutations) (Fig. 5B). We constructed a stacking ensemble model using these feature sets (Methods), which achieved the highest performance of 0.87 AUC (Fig. 5B). At a performance of 80% for correctly predicting non-responder status (specificity), the ensemble model had a 68% (CI: 55–81%) sensitivity for predicting responder status. Using cfDNA features based on chromatin accessibility sites from ATAC-seq data achieved only 0.72 AUC alone and did not improve the performance when included in the ensemble classifier (Supplementary Fig. 2C).

Given that an independent cohort of docetaxel-treated patients was not available for external validation in this proof-of-concept study, we applied the final model to predict response status in a cohort of 24 pre-treatment specimens from mCRPC patients planned to receive cabazitaxel (clinical characteristics in Supplementary Fig. 1B). Since cabazitaxel is only approved for treatment of patients who had previously received docetaxel, this would generally represent a docetaxel-resistant population, with the caveat that these patients could have had either intrinsic or acquired resistance to docetaxel. We focused on the ability of this model to correctly categorize these patients as non-responders to docetaxel (Methods). We achieved a concordance of 17.4 (72.4%), 18.0 (74.9%), 18.8 (78.5%), and 19.1 (79.6%) for (1) central coverage TFBS, (2) mean coverage TFBS, (3) CNAs, and (4) combined ensemble classifiers, respectively (Fig. 5C).

To determine what features were most impactful on the ensemble model’s predictions, we used SHapley Additive exPlanations (SHAP) analysis for the nucleosome accessibility and genomic alteration features (Fig. 5D, Supplementary Data 1-sheet 10). Among the features with the highest SHAP score, AR mutations were associated with increased likelihood of treatment response, whereas *TP53* mutations correlated with non-response to docetaxel. Additional features with high impact included nucleosome coverage for TFs NANOG, OSR2, ESR2, XBP1, GLIS3, HES2, and CNAs affecting chromosomal regions chr1p36.23, 1p31.3, and 2p16.3 (Fig. 5D).

## Discussion

Here, we analyze a cfDNA-based classifier for response and resistance to docetaxel chemotherapy in patients with mCRPC. Machine learning approaches have previously been reported for predicting response and resistance to docetaxel^44,45^, as well as to enzalutamide and abiraterone^46^ in prostate cancer. However, these approaches required the availability of tumor tissue for gene expression profiling^44^, whole transcriptome sequencing^46^, or assessment of non-coding RNAs^45^, which is not always clinically feasible and not easily tractable for analysis. We believe this is the first classifier reported for response prediction to docetaxel in mCRPC derived exclusively from non-invasive cfDNA sequencing.

An advantage of using machine learning techniques to generate a classifier is that individual features, which may not achieve statistical significance with regard to response prediction alone, can be leveraged in an ensemble method. On univariate analysis, only the *TP53* mutation was associated with docetaxel resistance, which is in line with previously published data^47^. That serves as an important positive control to demonstrate the validity of our unbiased techniques. None of the other mutations from targeted sequencing, nor any of the individual CNAs or TFBSs, were statistically significantly associated with response when accounting for FDR. Only when combined do these features demonstrate significant predictive ability.

Of note, while machine learning techniques can associate a set of features with an outcome of interest, each individual feature may not contribute functionally to the cancer phenotype. While *TP53* mutation is likely causal for docetaxel resistance, the other features may mark a state of “response” and “resistance” that would require further functional interrogation with regard to the biological underpinnings. Copy number analysis revealed trends towards more frequent deletions in docetaxel responders, as well as differences between groups in multiple loci of chromosomes 11 and 3. Notably, copy number losses of *YAP1* (11q22.1) and *TEAD1* (11p15.3) were enriched in responders to docetaxel, which is consistent with prior studies implicating the Hippo pathway in docetaxel resistance^48,49^.

However, in our study, higher nucleosome occupancy of NANOG (suggesting lower activity) was associated with non-response to docetaxel, which is inconsistent with prior studies suggesting an association of higher NANOG expression with chemoresistance in breast^50^ and prostate^51^ cancer models. Another study reported that NANOG is a direct AR target, and while its overexpression increased proliferation of prostate cancer cells, it did not promote docetaxel resistance^52^. These discrepant findings highlight caveats in interpreting results from Griffin, including the possibility that NANOG activity may not directly correlate with expression and that it may share binding sites with other TFs. As such, it is not clear whether low NANOG activity is functionally related to docetaxel resistance or is a marker of a resistant state in patients. In other cases, the observed associations align with pre-clinical data and may be functionally relevant. For example, in our study, lower nucleosome occupancy (i.e., higher activity) of serum response factor (SRF) was associated with non-response to docetaxel, which is concordant with prior findings where this transcription factor was associated with docetaxel resistance, and siRNA-mediated functional knockdown of SRF led to a reversal of resistance to docetaxel treatment in the docetaxel-resistant PC-3 cell line model^53^. In any case, these analyses nominate several novel transcription factors associated with docetaxel response or resistance for functional validation in model systems.

The use of multi-modal data has already demonstrated clinical utility in predicting treatment benefit, even without a full mechanistic understanding of the underlying features. A notable example is the ArteraAI biomarker test, which combines clinical features with imaging features extracted from hematoxylin and eosin-stained slides^54^ to derive a score that has been demonstrated to predict benefit from the addition of androgen deprivation therapy to radiation therapy for prostate cancer treatment^55^. In our analysis, while individual classifiers based solely on mutations, CNAs, transcription factor mean coverage, or transcription factor central coverage each showed predictive ability for docetaxel response, combining these features yielded only modest performance improvements. This suggests substantial overlap between these feature sets, indicating they may capture similar underlying biological processes rather than providing independent predictive information.

Strengths of our study include the utilization of banked specimens to reflect a “real world” population of participants treated per standard of care, and the use of unbiased analytic tools to associate the identified features with clinical outcomes abstracted through manual medical record review, which may not be feasible in larger cohorts. However, a limitation of this study is the relatively modest sample size of the cohort and the absence of an independent validation cohort including docetaxel responders. In addition, the sequencing metrics required for Griffin analysis also limit the applicability of this tool. Within these limitations, our findings are robust in internal cross-validation and when applied to pre-cabazitaxel specimens as an external “docetaxel-resistant” validation set, understanding that patients planned for cabazitaxel represent a mixed population of those with both intrinsic and acquired resistance to docetaxel. While a tumor fraction requirement of ≥ 0.05 can be limiting in practice, given the expanding number of treatment options in mCRPC, many patients will have relatively high burden disease (and thus high TFx) by the time docetaxel would be considered as a treatment option. Another limitation of this study is whether the definition of response based on PSA50 is an appropriate indicator of clinical benefit, but this is a commonly used parameter in prostate cancer clinical trials and is more tractable for extraction from the medical record as compared to other clinical outcomes (e.g., radiographic progression-free survival).

Overall, this study demonstrates an important proof-of-concept around the potential utility of multi-modal circulating cell-free analysis for the prediction of treatment response. This classifier will need to be validated in larger cohorts to demonstrate clinical utility. If validated, such a tool would have an immediate impact on clinical decision-making in recommending docetaxel over other potentially active agents in taxane-naïve mCRPC, such as lutetium Lu 177 vipivotide tetraxetan^19^ or radium-223^15,18^. In addition, we have nominated a set of transcription factors for functional interrogation with regard to docetaxel sensitivity and resistance in model systems. Integrating deeper whole genome sequencing and direct epigenomic^56^ profiling may further enhance the predictive performance of our models. While this classifier focuses on distinguishing responders from those with intrinsic resistance, paired comparisons from specimens acquired pre-treatment and at progression could better characterize mechanisms of acquired resistance. Further studies will be required to demonstrate applicability to other tumor types and treatment modalities.

## Methods

### Sex as a biological variable

All study participants were biological males.

### Human subjects

Eligible mCRPC patients were identified through the Institutional Review Board (IRB) approved Prostate Clinical Research Information System (CRIS) database at Dana–Farber Cancer Institute^57^. The CRIS system comprises data-entry software, a central data repository, prospective collection of patient data, including comprehensive follow-up of all patients, and tightly integrated security measures, as previously described^57^. Patients were included in the “pre-docetaxel” cohort if they had at least 1 plasma sample collected and stored within 12 months prior to initiating docetaxel for mCRPC (between 2001 and 2016) and were included in the “pre-cabazitaxel” cohort if they had at least 1 plasma sample collected and stored within 12 months prior to starting cabazitaxel for mCRPC (between 2010 and 2016). Patients who received a taxane for hormone-sensitive prostate cancer (biochemical recurrence or metastatic hormone-sensitive prostate cancer), cabazitaxel before docetaxel, or any taxane in combination with other agents were excluded.

### Study approval

The present studies in humans were reviewed and approved by the IRB of the Dana–Farber/Harvard Cancer Center, Boston, Massachusetts. This study was conducted in accordance with the Declaration of Helsinki and International Council of Harmonisation Good Clinical Practice Guidelines, and other applicable guidelines, laws, and regulations. All patients had consented to Dana–Farber/Harvard Cancer Center protocol no. 01-045 “Collection of Specimens and Clinical Data for Patients with Prostate cancer or at High Risk for Prostate Cancer.” This protocol allows for banking of tissue and blood specimens for research use, including comprehensive genetic sequencing and data sharing. Medical record review was approved by the IRB per DF/HCC Protocol #18-135.

### Clinical specimens

Plasma specimens banked under protocol #01-045 were collected in EDTA (BD) tubes and were processed to component plasma, buffy coat, and erythrocytes from blood using standard methods at the time of collection (i.e., with a single high-speed spin and with an uncertain gap between blood collection and plasma processing up to ~8 h) and subsequently frozen.

The identified banked plasma samples (1000 μL/subject) were retrieved from the genitourinary Gelb tumor bank. These frozen aliquots of plasma were thawed at room temperature and then subjected to high-speed spin. The Qiagen Circulating DNA kit (QIAGEN, Germantown, MD, USA) on the QIAsymphony liquid handling system was used to extract the cfDNA from the plasma samples.

### Ultra low-pass whole-genome sequencing

ULP-WGS was performed on the extracted cfDNA, and sequencing information was run through ichorCNA to detect cases harboring detectable tumor DNA content and CNAs. In detail, the isolated cfDNA was quantified using the PicoGreen (Life Science Technologies, Waltham, MA, USA) assay on a Hamilton STAR-line liquid handling system. CfDNA sequencing libraries were constructed using the Kapa Hyper Prep kit with custom adapters (Integrated DNA Technologies, Coralville, IA, USA). A median of 5 ng of cfDNA input (3–20 ng) was used for ULP-WGS, which was performed using a Hamilton STAR-line liquid handling system (Hamilton Company, Reno, NV, USA). Constructed sequencing libraries were pooled (2 μL of each × 96 per pool) and sequenced using 100 bp paired-end runs over 1× lane on a HiSeq2500 (Illumina, San Diego, CA, USA) for ULP-WGS (~0.1× coverage).

### Analysis of ultra low-pass whole-genome sequencing data

ULP-WGS of cfDNA was performed to average genome-wide fold coverage of 0.1X. Segment copy number and TFx were derived via ichorCNA^24^ using the standard configurations in the pipeline accessible at https://github.com/GavinHaLab/ichorCNA/releases/tag/v0.4.0. Briefly, the genome was divided into non-overlapping windows, or bins, of 1 Mb, and aligned reads were counted based on overlap within each bin. Centromeres were filtered based on chromosome gap coordinates obtained from UCSC for hg38, including a padding of 1 Mb bin up- and downstream of the gap. The read counts were then normalized to correct for GC-content and mappability biases. Log2 copy ratios were computed for each bin relative to a reference panel of normals generated from ULP-WGS data of 27 healthy donors. Discrete copy number prediction and TFx estimation were derived using a hidden Markov model. The copy number states can be mapped to integer values corresponding to hemizygous deletions (HETD, 1), copy neutral (NEUT, 2), copy gain (GAIN, 3), amplification (AMP, 4), and high-level amplification (HLAMP, 5). Of note, homozygous deletion states typically occur at smaller scales than the bin sizes used for the analysis and were therefore excluded. Genome-wide copy number plots were generated by ichorCNA.

### Plasma cell-free DNA-targeted panel sequencing

For participants with a “pre-docetaxel” cfDNA specimen with TFx > 0.04, the same library constructed for ULP-WGS underwent hybrid capture for 319 prostate cancer-relevant genes. Hybridization and capture are performed using the relevant components of IDT’s XGen hybridization and wash kit and following the manufacturer’s suggested protocol, with the exception of using custom panels synthesized by TWIST Biosciences instead of the recommended xGen Lockdown Panels and Probe Pools. A set of 12-plex prehybridization pools was created. These prehybridization pools were created by equivolume pooling of the normalized libraries, IDT XGen blocking oligos, and human Cot-1, included in the IDT xGen Hybridization and Wash Kit (IDTDNA catalog no 1080584). The prehybridization pools underwent lyophilization using the Biotage SPE-DRY concentrator. After lyophilization, custom exome bait (TWIST Biosciences, Supplementary Data 2–4), along with hybridization mastermix, was added to the lyophilized pool prior to resuspension. Samples are incubated overnight at 65°C using an Eppendorf Mastercycler x50. Library normalization and hybridization setup were performed on a Hamilton Starlet liquid handling platform, whereas target capture was performed on the Agilent Bravo automated platform. After capture, a PCR was performed to amplify the capture material using custom primers developed by IDT (Dual Index Universal Primer Forward: AATGATACGGCGACCACCGAGATCTCAC; Dual Index Universal Primer Reverse: CAAGCAGAAGACGGCATACGAGAT). After capture enrichment, library pools were quantified using qPCR (automated assay on the Agilent Bravo), using the KAPA Library Quantification kit (Illumina catalog no. 07960204001) with probes specific to the ends of the adapters. On the basis of qPCR quantification, pools were normalized using a Hamilton Starlet to 2 nmol/L and sequenced using Illumina sequencing technology. Cluster amplification of library pools was performed according to the manufacturer’s protocol (Illumina) using Exclusion Amplification cluster chemistry and HiSeq X flow cells. Flowcells were sequenced on v2 Sequencing-by-Synthesis chemistry for HiSeq X flowcells. The flow cells are then analyzed using RTA v.2.7.3 or later. Each pool of libraries was run on paired 151 bp runs, reading the dual-indexed sequences to identify molecular indices, and sequenced across the number of lanes needed to meet coverage for all libraries in the pool. Reads were aligned using BWA-MEM. Consensus duplex sequences were generated from reads using fgbio (http://fulcrumgenomics.github.io/fgbio/) CallDuplexConsensusReads v1.0.0 with allowable UMI distance of 1. FilterConsensusReads was utilized with --max-read-error-rate 0.01, --max-base-error-rate 0.05, --min-mean-base-quality 40, --require-single-strand-agreement true. Next, we used Mutect2 from GATK v4.1.8.1 to identify single-nucleotide variants and short indels with --read-filter NotSupplementaryAlignmentReadFilter, --dont-use-soft-clipped-bases true, --default-af 0.001, --tumor-lod-to-emit 0, --max-reads-per-alignment-start 0, --initial-tumor-lod 0, --pcr-snv-qual 70, --pcr-indel-qual 70^58^. Variants were annotated using Funcotator from GATK v4.1.8.1 (https://gatk.broadinstitute.org/hc/en-us/articles/360037224432-Funcotator). Nonsense mutations and indels were considered deleterious for tumor suppressors, as were missense mutations categorized as significant, by COSMIC Cancer Mutation Census release v98 (https://cancer.sanger.ac.uk/cmc/home).

For CNA analysis, the GATK v4.0.5.1 CNV pipeline (https://github.com/gatk-workflows/gatk4-somatic-cnvs) was applied to the targeted panel sequencing data. A panel of normals (PoN) consisting of 270 unrelated blood normal germline samples was also sequenced with this same custom panel workflow. Copy number segment files output by the CNV pipeline were used. Absolute copy number was corrected based on tumor purity using the formula: ACN = 2 × [(CR−1)/TF + 1], where ACN = absolute copy number, CR = copy ratio, TF = tumor fraction from ichorCNA estimated on ULP-WGS for the same sample. Copy number categories were determined as follows:

- Amplifications were called if a sample had TF ≥ 0.1 and ACN ≥ 5
- Deletions were called if a sample had TF ≥ 0.3 and ACN ≤ 0.5

A stricter cut-off was used for deletions due to challenges in detecting coverage losses for samples with low tumor purity. Thus, we recognize that some deletion events may be missed in the targeted panel data.

### Germline variant calling

Germline variants were determined using DeepVariant v0.8.0^59^. Variants identified were considered deleterious if listed as “pathogenic” or “likely pathogenic” based on ClinVar (https://www.ncbi.nlm.nih.gov/clinvar/) annotations via Funcotator (https://gatk.broadinstitute.org/hc/en-us/articles/360037224432-Funcotator).

### Nucleosome accessibility profiling

BAM files from docetaxel-treated (*n* = 46) and cabazitaxel-treated (*n* = 28) samples, obtained through ultra-low-pass whole-genome sequencing (ULP-WGS), were analyzed using Griffin^33^. Griffin infers chromatin accessibility and nucleosome occupancy analysis from cfDNA sequencing data as low as 0.1× genome coverage, incorporating a fragment-level GC-bias correction.

Griffin was applied to analyze known sites of interest to infer transcription factor (TF) activity. The standard workflow for Griffin v0.2.0 was used (https://github.com/GavinHaLab/Griffin). Briefly, TFBSs were downloaded from the GTRD database^60^ and TFs with less than 10,000 sites on autosomes were excluded, resulting in 377 TFs. For each remaining TF, the top 10,000 sites were selected by choosing those with the highest “peak.count” (number of times that peak has been observed across all experiments). For nucleosome profile quantification, three features were extracted: (1) central coverage at the site (±30 bp) and (2) mean coverage across ±1000 bp. These metrics were used to assess chromatin accessibility and nucleosome occupancy.

### Differential binding analysis of PDX Model ATAC-seq data

Assessed from GSE298042, 31 samples from PDX models (17 responders, 14 non-responders) underwent assay for transposase-accessible chromatin with sequencing (ATAC-Seq). Fastq files were aligned to hg38 using bowtie2 v2.4.2^61^. PCR duplicates were marked and removed using Picard MarkDuplicates v2.24.1 (https://broadinstitute.github.io/picard/). Peaks were called using Genrich v0.6.1 in ATAC-Seq mode (https://github.com/jsh58/Genrich?tab=readme-ov-file#atacseq). Called peaks had the ENCODE blacklisted regions^62^ removed.

Differential analysis was performed using DiffBind 3.10.1^63^ using default settings. Differential regions were filtered by a Benjamini-Hochberg FDR cut-off of 0.05. These sites were further filtered to “minor” differential sites with a Log Fold of ±1 and “major” differential sites with a Log Fold of ±2.

### Phasing analysis in cfDNA using TritonNP and ATAC-seq

ATAC-seq analysis was conducted to assess nucleosome periodicity in cfDNA and its association with transcriptional activity across different CRPC phenotypes. We utilized TritonNP, a recently published tool designed for quantifying inter-nucleosomal spacing and predicting nucleosome phasing and periodicity^34^. This methodology allows for the identification of phased nucleosome dyad positions and provides an average inter-nucleosome distance, reflecting nucleosome occupancy and stability within the gene body.

### Copy number alteration analysis (ULP-WGS)

CNAs were analyzed using the ichorCNA software^24^, as described above (“Analysis of ultra low-pass whole-genome sequencing data”). CNA profiles were analyzed at three different genomic scales: chromosome arms, cytobands, and genes.

Mean copy number differences for each chromosome arm were calculated between responders and non-responders. Statistical significance was determined using the Mann–Whitney *U* test. Chromosome arms were further stratified into four copy number ranks: deletion (≤0.5), neutral (>0.5 and <1.5), and gain (≥1.5). Frequencies of each rank were compared between groups using Chi-square and Fisher’s exact tests, with significant differences highlighted (*p* < 0.05).

Cytoband-level CNAs were identified by segmenting the genome into bands and comparing copy number frequencies between groups. Fisher’s exact tests were used to detect significantly altered bands (*p* < 0.05), and Chi-square tests assessed the distribution of deletions, amplifications, and other copy number ranks. Cytobands with recurrent CNAs were annotated and integrated into downstream analyses.

Gene-level CNAs were determined by mapping genomic segments to individual genes. A total of 765 genes implicated in docetaxel resistance, representing key protein families and pathways, were preselected based on prior literature. Fisher’s exact tests identified genes with significant copy number differences between responders and non-responders.

### Docetaxel treatment response classifier

We employed the XGBoost 1.6.2 binary classification algorithm to predict docetaxel treatment response vs non-response. XGBoost (eXtreme Gradient Boosting) is a scalable and efficient machine learning algorithm that utilizes gradient-boosted decision trees to optimize predictive performance. Separate models were independently trained on: (1) central and (2) mean TF binding site coverage values (379 features each), (3) CNAs at the chromosome band level (696 features), and (4) mutation status (9 features) across all 46 patients treated with docetaxel, using a binary target variable indicating treatment response or non-response.

A combined ensemble model was constructed using a Stacking Classifier (scikit-learn 1.4.0) to integrate predictions from four independently trained XGB classifiers, each trained on a subset of features. The Stacking Classifier, as implemented in the sklearn library, is an ensemble learning method that combines the predictions of multiple base estimators through a meta-classifier. A logistic regression model was selected for its ability to effectively integrate probabilistic outputs and provide well-calibrated final predictions. In this approach, the outputs (predicted probabilities) of the base models serve as input features for the meta-classifier, which is trained to optimize overall predictive performance.

Hyperparameter optimization was conducted using grid search to enhance model performance. The final optimized parameters for the XGB classifiers included the learning rate, maximum depth, number of estimators, and minimum child weight. Model evaluation was performed using a cross-validation set consisting exclusively of docetaxel-treated patients to tune and validate the models. Subsequently, the optimized models were tested on a separate test set comprising cabazitaxel-treated patients to assess their generalizability to an independent cohort.

Performance metrics such as accuracy, precision, recall, F1 score, and area under the receiver operating characteristic curve (AUROC) were utilized to evaluate predictive capacity. Cross-validation ensured robust internal validation, minimizing overfitting and providing reliable estimates of model performance on unseen data. The test set evaluation provided an independent assessment of the models’ ability to generalize across treatment contexts. Feature importance was analyzed using SHapley Additive exPlanations (SHAP ver. 0.41.0), allowing for the identification of key predictors contributing to the model’s decision-making process.

## Supplementary information

Supplementary Information

Supplementary Data1

Supplementary Data2

Supplementary Data3

Supplementary Data4

## Data Availability

Sequencing data available upon request under dbGaP accession code phs001417_v3.

## Code availability

Custom scripts used for this study can be accessed at https://github.com/GavinHaLab/CRPC-docetaxel-cfDNA-paper.
