# Supplementary material for: Multi-modal circulating cell-free DNA profiling to predict response to docetaxel in metastatic castration-resistant prostate cancer
Source: NPJ Precis Oncol. 2026 Apr 28;10:255. doi: 10.1038/s41698-026-01454-6 (PMC13328454; doi:10.1038/s41698-026-01454-6)
Supplement: Supplementary file 1 — Supplementary Information [file 41698_2026_1454_MOESM1_ESM.pdf]

A

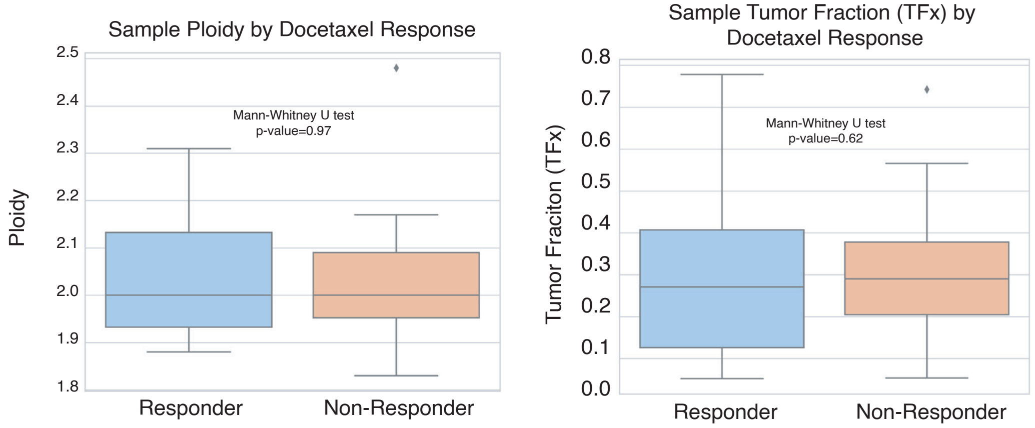

B

Characteristic	Median (IQR)	Missing (n)
Age	68.0 (60.0 - 74.2)	0
Initial PSA (ng/mL)	98.6 (47.2 - 297.6)	0
Prior number of Systemic Therapies	4 (3 - 5)	0
Days Betweeen ctDNA and Cabazitaxel Initiation	150 (65 - 223)	0
Number of Cycles of Cabazitaxel	4 (3 - 6)	1

Supplementary Figure 1. Additional sample information and clinical characteristics. A) Overall ploidy and tumor fraction, as determined by ichorCNA, are not statistically significant between responders and non-responders for the pre-docetaxel cohort. **B)** Clinical characteristics of the pre-cabazitaxel cohort which was used as the validation set in classification of docetaxel response (Figure 5C).

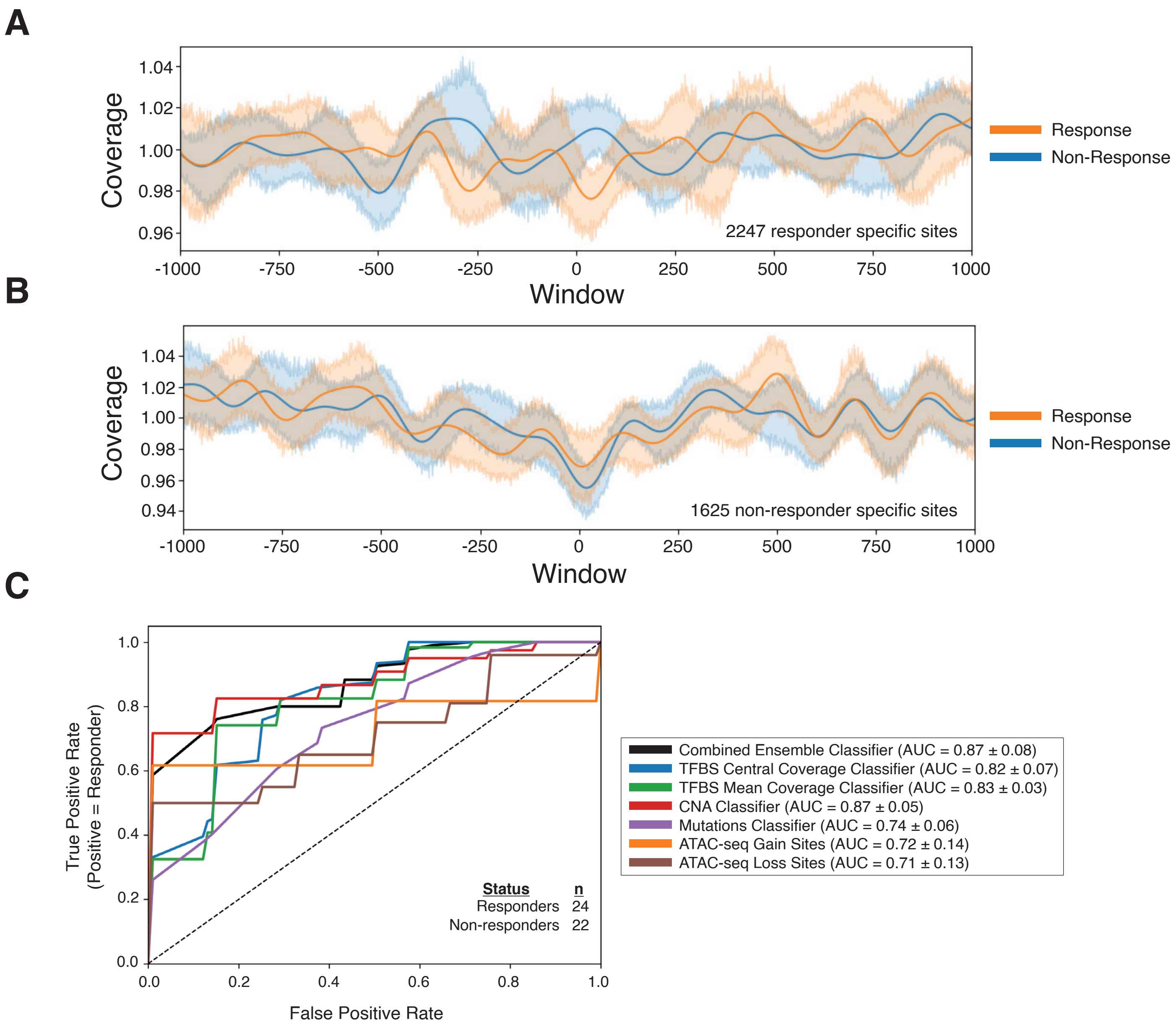

Supplementary Figure 2. Incorporation of ATAQ-seq Data. TritonNP is a tool for analyzing chromatin accessibility and nucleosome positioning from patient ATAC-seq data. TritonNP-derived signal profiles representing nucleosome centers and coverage depths of **A)** 2,247 responder-specific sites and **B)** 1,625 non-responder-specific sites, both derived from differential binding analysis of PDX models (Methods). **C)** Incorporation of ATAQ-seq 'gain' (orange line) and 'loss' (brown line) sites into classification model (similar to Figure 5B), demonstrating modest performance individually but not improving combined ensemble performance.

Supplementary Data 1. Contains an Excel workbook with ten sheets of data referenced throughout the manuscript. Sheet 1 includes patient information such as PSA levels, TFx, and ploidy. Sheets 2 and 3 present Fisher's Exact Test p-values for copy number alterations (CNAs), analyzed at the chromosome arm and cytoband levels, respectively, comparing deletions, neutral states, and gains. Sheets 4 and 5 contain Chi-Square Test p-values at the gene level for copy number loss and gain, respectively. Sheets 6 and 7 list significantly enriched gene pathways associated with copy number loss and gain. Sheets 8 and 9 provide transcription factor binding site data from Griffin analysis, including central and mean coverage values with corresponding statistical test p-values. Sheet 10 summarizes SHAP values for the top 75 features in the pre-docetaxel patient cohort.

Supplementary Data 2. List of genes and intergenic regions included in the prostate cancer-specific bait set for targeted panel sequencing.

Supplementary Data 3. Interval list of prostate cancer-specific bait set.

Supplementary Data 4. Interval list of coverage regions for prostate cancer-specific bait set.
